# Characterization and Antiproliferative Activity of a Novel 2-Aminothiophene Derivative-β-Cyclodextrin Binary System

**DOI:** 10.3390/molecules23123130

**Published:** 2018-11-29

**Authors:** Elayne Barros Ferreira, Walter Ferreira da Silva Júnior, Jonas Gabriel de Oliveira Pinheiro, Aldilane Gonçalves da Fonseca, Telma Maria Araújo Moura Lemos, Hugo Alexandre de Oliveira Rocha, Eduardo Pereira de Azevedo, Francisco Jaime Bezerra Mendonça Junior, Ádley Antonini Neves de Lima

**Affiliations:** 1 Department of Pharmacy, Federal University of Rio Grande do Norte, Av. General Cordeiro de Farias, s/n, Petrópolis, Natal, Rio Grande do Norte 59012-570, Brazil; elayne.barros@hotmail.com (E.B.F.); walterjuniornt@hotmail.com (W.F.d.S.J.); jgopinheiro@gmail.com (J.G.d.O.P.); 2 Department of Clinical and Toxicological Analysis, Federal University of Rio Grande do Norte, Av. General Cordeiro de Faria, s/n, Petrópolis, Natal, Rio Grande do Norte 59012-570, Brazil; aldilanefonseca@hotmail.com (A.G.d.F.); telmaml@yahoo.com.br (T.M.A.M.L.); 3 Department of Biochemistry, Federal University of Rio Grande do Norte, Av. Senador Salgado Filho, 3000, Lagoa Nova, Natal, Rio Grande do Norte 59078-970, Brazil; hugo@cb.ufrn.br; 4 Graduate Program of Biotechnology, Laureate International Universities—Universidade Potiguar (UnP), Av. Sen. Salgado Filho, 1610, Lagoa Nova, Natal, Rio Grande do Norte 59056-000, Brazil; azevedoep@hotmail.com; 5 Department of Biological Sciences, State University of Paraíba, Rua Horácio Trajano de Oliveira, s/n, Cristo Redentor, João Pessoa 58071-160, Brazil; xicolouco@hotmail.com

**Keywords:** 2-aminothiophene derivative, β-cyclodextrin, antitumor, anti-proliferative, cytostatic

## Abstract

The novel 2-aminothiophene derivative 2-amino-4,5,6,7-tetrahydrobenzo[*b*]thiophene-3-carbonitrile (6CN) has shown potential anti-proliferative activity in human cancer cell lines. However, the poor aqueous solubility of 6CN impairs its clinical use. This work aimed to develop binary 6CN-β-cyclodextrin (βCD) systems with the purpose of increasing 6CN solubility in water and therefore, to improve its pharmacological activity. The 6CN-βCD binary systems were prepared by physical mixing, kneading and rotary evaporation methods and further characterized by FTIR, XRD, DSC, TG and SEM. In addition, molecular modeling and phase solubility studies were performed. Finally, MTT assays were performed to investigate the cytostatic and anti-proliferative effects of 6CN-βCD binary systems. The characterization results show evident changes in the physicochemical properties of 6CN after the formation of the binary systems with βCD. In addition, 6CN was associated with βCD in aqueous solution and the solid state, which was confirmed by molecular modeling and the aforementioned characterization techniques. Phase solubility studies indicated that βCD forms stable 1:1 complexes with 6CN. The MTT assay demonstrated the cytostatic and anti-proliferative activities of 6CN-βCD binary systems and therefore, these might be considered as promising candidates for new anticancer drugs.

## 1. Introduction

**Figure 1:**
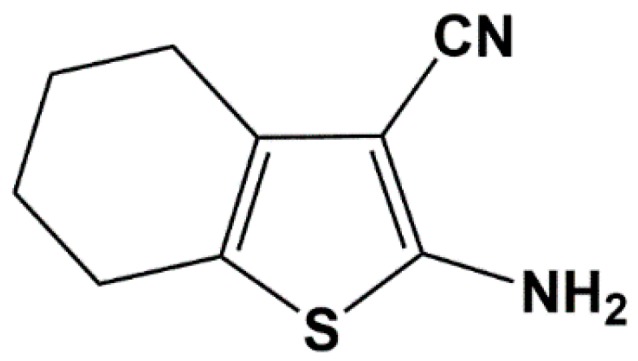
Chemical structure of 2-amino-4,5,6,7-tetrahydrobenzo[*b*]thiophene-3-carbonitrile (6CN).

Thiophenes belong to a group of aromatic heterocyclic compounds that occupy a special position among the new chemical entities due to the innovations in their synthesis in addition to their chemical stability and structural simplicity. Moreover, thiophenes have a wide spectrum of pharmacological properties that include antimicrobial, anti-inflammatory, antifungal, antioxidant, antiplatelet [1] and antileishmanial activities [2]. Recent reports have shown that 2-aminothiophene derivatives have anti-proliferative activity against human and pancreatic cervical cells and therefore, might act as cytostatic agents. The potential application of 2-aminothiophene derivatives in cancer therapy is of great clinical interest and they may be considered promising anticancer agents [1]. The compound 2-amino-4,5,6,7-tetrahydrobenzo[*b*]thiophene-3-carbonitrile (6CN, Figure 1) is a new prototype that belongs to the class of 2-aminothiophene derivatives.

Like most new chemical entities (NCEs), 6CN presents low solubility in water, which impairs its clinical use due to poor bioavailability, especially through the oral route [3]. The development of binary systems of NCEs and cyclodextrins (CDs) has shown to be an effective alternative to improve the aqueous solubility of these molecules [4].

CDs are a class of pharmaceutical excipients that contain D-glucopyranose units linked together to form cyclic conical structures. In the pharmaceutical field, cyclodextrins have been extensively used to improve the solubility, stability and bioavailability of poorly soluble drugs [5]. Among the different cyclodextrins, β-cyclodextrin (βCD) is the most commonly used one in the pharmaceutical industry due to the adequate size of its internal cavity, which allows the inclusion of most aromatic compounds [6,7]

The discovery of anticancer agents that combine efficacy, safety and convenience remains a challenge for the scientific community. Currently, the development of anticancer chemotherapy is conducted by identifying cytotoxic compounds that are capable of killing cancer cells [1]. Although these cytotoxic agents may be able to control tumor growth, the lack of specificity of such agents compromises their efficacy and may lead to various complications such as toxicity and severe side effects [8].

Hepatic adenocarcinoma, which accounts for 70% to 85% of the total liver cancers around the world, is considered one of the most aggressive cancers [9,10], and although renal carcinoma is relatively common, the chances of cure are fairly low [11].

The aim of this current work was to obtain binary systems of 6CN with βCD, as well as to characterize the 6CN-βCD systems by FTIR, XRD, DSC, TG and SEM. In addition, molecular modeling and phase solubility studies were performed. Finally, the anti-proliferative activity of the 6CN-βCD binary systems against normal fibroblast (3T3), hepatic adenocarcinoma (HepG2) and renal carcinoma (786-0) cell lines were investigated.

## 2. Results and Discussion

### 2.1. Molecular Modeling and Docking Studies

**Figure 2:**
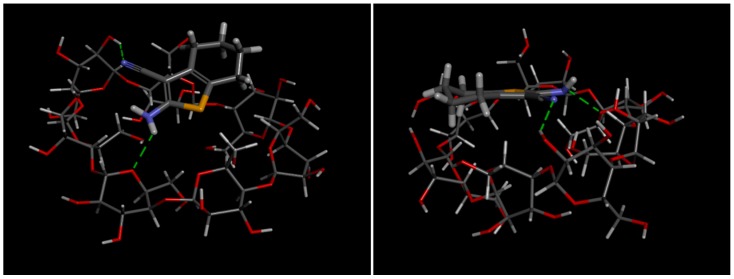
3D demonstration of 6CN-βCD association at its lower energy at two different angles. Dashed lines represent intermolecular hydrogen bonds. The chemical elements are represented by different colors: yellow (sulfur), blue (nitrogen), red (oxygen) and gray (carbon).

The molecular docking performed through Autodock 4.2 indicates that 6CN forms a stable association with βCD, herein referred as the binary system. Figure 2 shows the 3D structure of this complex at the lowest energy state viewed from two different angles, as well as the main chemical interactions that stabilize the association between 6CN and βCD.

The affinity energy of this binary system was −4.3 kcal/mol. Green dashed lines represent intermolecular hydrogen bonds between 6CN and βCD, which are the strongest interactions that contribute to the stabilization of the 6CN-βCD association. The groups responsible for these chemical interactions are amine (–NH_2_) and nitrile (C≡N), both from 6CN, which form hydrogen bonds with the lactol endocyclic oxygen and the hydroxyl group at position 2 of βCD, respectively.

The computational molecular modeling and docking were carried out with the purpose of obtaining information about the three-dimensional geometry of a possible association between 6CN and βCD. In addition, these studies aim to elucidate the energy affinity and the forces involved in the stabilization of these associations. The affinity energy of the 6CN-βCD binary system (−4.3 kcal/mol) indicates an energetically favorable interaction between 6CN and βCD (Eleamen et al., [12]). Our results suggest that the preferential interaction between 6CN and βCD occurs through the outer part of the latter, as observed by Eleamen et al. [12], whose work studied another 2-aminothiophene derivative.

### 2.2. Phase Diagram Study

**Figure 3:**
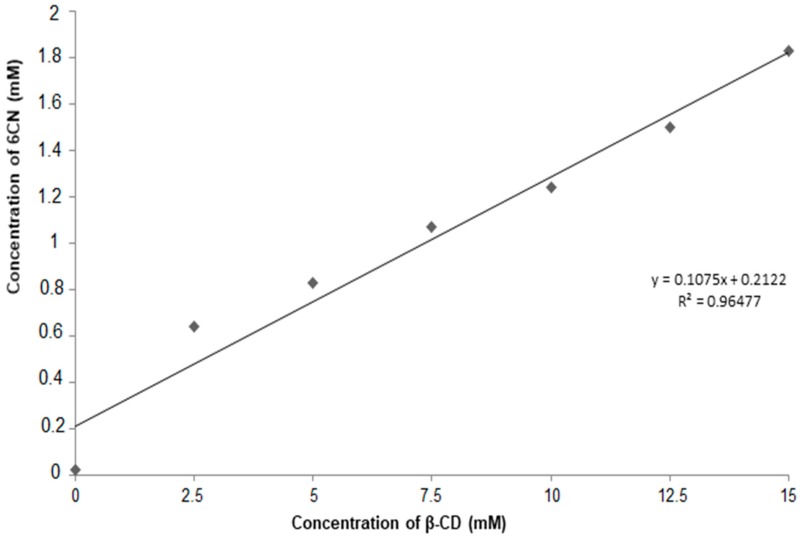
Phase solubility diagram of 6CN with different concentrations of βCD.

Based on the method described by Higuchi and Connors [13], the phase solubility diagram was obtained for 6CN with different concentrations of βCD, as shown in Figure 3. Since phase diagrams provide evidences of the stoichiometry between drug and cyclodextrin, the phase solubility study is usually the first evidence of an association between cyclodextrin and a drug. The linear equation y = 0.1075x + 0.2122 was then obtained with a linear correlation coefficient (R²) of 0.9648.

According to the classification of Higuchi and Connors, the diagram shown in Figure 3 is classified as type A, which corresponds to the formation of soluble complexes. Therefore, an increase in the solubility of 6CN as a function of βCD concentration was observed in this study. In addition, according to the nature of the 6CN-βCD association and due to the linearity of the phase diagram, it can be classified as subtype AL.

The strength or magnitude of the drug association with cyclodextrins can be described by the stability constant (KS), which can be calculated from the initial linear portion of the phase solubility diagram [14] using the following equation:

Ks = slope/S_0_ (1 − slope) (1)

where S_0_ is the solubility of 6CN in the absence of CD (S_0_ = 0.027) and ‘slope’ corresponds to the slope of the curve (0.1075). After adequate mathematical treatment, the KS of 6CN in the presence of βCD was calculated as 446. The Gibbs free energy change (ΔG) involved in the association process was calculated from the stability constant (KS) according to the following equation:

ΔG = −RT InK5(2)

where R represents the universal constant of the perfect gases (8.314 J) and T is the experimental temperature (298 K). After adequate mathematical treatment, the obtained ΔG was −15.1 KJ/mol.

Phase solubility diagrams are dependent upon the equilibrium model that is established during the association between drug and cyclodextrin, where they are classified into types and subtypes. Since the slope of the curve is less than 1 (0.1075), it is generally assumed that the stoichiometry of the 6CN-βCD association is 1:1 and that the complexation efficiency is less than 100% [15]. In binary systems, 1:1 is the most common molar ratio between drug and cyclodextrin, although ratios of 2:1, 1:2, 2:2 are also observed. However, depending on the presence of additional molecules and on the size of the drug, ternary complexes can be formed, where association constants that are different from those of the binary systems are observed [16]. KS values between 200 and 5000 M^−1^ are associated with improvement in both stability and solubility of drugs [17,18]. Thus, the 6CN-βCD binary system can in fact enhance the solubility and stability of 6CN. The negative sign of ΔG indicates a spontaneous association between 6CN and βCD in aqueous medium [19].

### 2.3. Differential Scanning Calorimetry (DSC)

**Figure 4:**
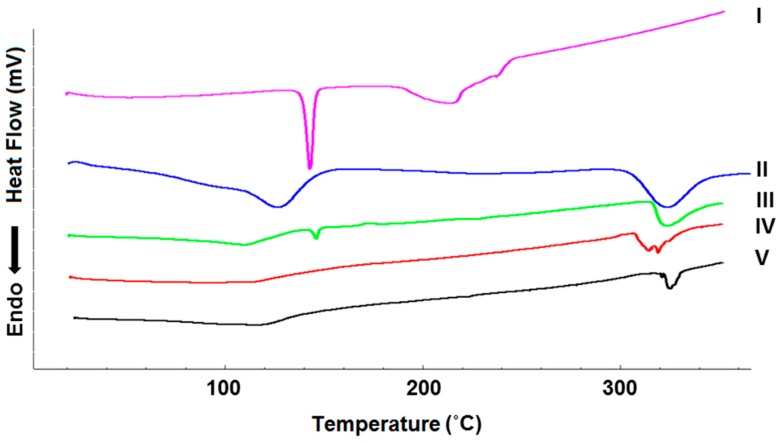
DSC curves for 6CN (I), βCD (II) and 6CN-βCD obtained by PM (III), KND (IV) and ROTA (V). The DSC curves were obtained at a temperature range of 25 to 350 °C, at a heating rate of 10 °C min^−1^, with dynamic atmosphere of N_2_ (50 mL min^−1^).

The DSC curves for 6CN, βCD and the binary systems are shown in Figure 4. The curve corresponding to 6CN shows two endothermic events, the first one (145–151 °C) is characteristic of 6CN melting (T_onset_ of 145 °C, T_endset_ of 151 °C, T_peak_ of 148 °C and ΔH −113 J g^−1^), which characterizes a crystalline compound due to its well-defined endothermic event.

The second event occurs between 188 and 253 °C (T_onset_ of 188 °C, T_endset_ of 253 °C, T_peak_ of 217 °C and ΔH −189.66 J g^−1^), which seems to be attributable to the degradation of 6CN.

The DSC curve for βCD shows that the first event occurs between 110 and 137 °C (T_onset_ of 110 °C, T_endset_ of 137 °C, T_peak_ 115 °C and ΔH −13.22 J g^−1^), which relates to the loss of water from the βCD cavity. The second event corresponds to the decomposition of βCD, which occurs between 306 and 340 °C (T_onset_ of 306 °C, T_endset_ of 340 °C, T_peak_ of 323 °C and ΔH −231.82 J g^−1^).

The DSC curve for the physical mixture (PM) shows an event between 143 and 147 °C, (T_onset_ of 143 °C, T_endset_ of 147 °C, T_peak_ of 146 °C and ΔH −9,08 J g^−1^). The curve profile is similar to that observed for both individual components, where the thermal events related to 6CN and βCD are still evident. Therefore, due to the lack of changes in the thermal events of 6CN and βCD in the PM, it can be inferred that no complexation took place between both compounds [19].

On the other hand, the DSC curves for the binary systems obtained by kneading (KND) and rotary evaporation (ROTA) show that the characteristic endothermic events of 6CN disappeared, which seems to indicate that this drug is no longer in its crystalline form. Since the thermal profile of βCD can be observed in the DSC curves for KND and ROTA, it seems obvious to infer that 6CN may be associated with βCD.

The second thermal event observed on the DSC curve for 6CN (188–253 °C) was absent in the curves for the binary mixtures (PM, KND and ROTA). On the other hand, it seems that this endothermic event, which is attributed to the decomposition of 6CN, has been shifted to 306–340 °C, which is close to the thermal event attributed to βCD. Such an increase in the decomposition temperature of 6CN usually occurs when the sample exists in the complexed state, which seems to indicate that an association between 6CN and βCD has taken place.

The thermal analyses were primarily used to compare the thermal behavior of each individual component of the binary system with that of the 6CN-βCD. As a rule of thumb, the association between drug and cyclodextrins is characterized by changes in DSC profiles of the drug alone and that of the drug-cyclodextrin binary system [15]. Therefore, DSC has been successfully used as an efficient tool to evidence associations between drugs and cyclodextrins. The rationale behind this technique is that the melting point of the host molecule usually changes to a different temperature or even disappears when it is associated with cyclodextrin [20]. Therefore, since the thermograms of the binary systems show the disappearance of the 6CN’s melting peak as well as the displacement of the 6CN decomposition peak, it suggests that this drug is associated with βCD [19,21,22].

### 2.4. Thermogravimetry Analysis (TGA)

**Figure 5:**
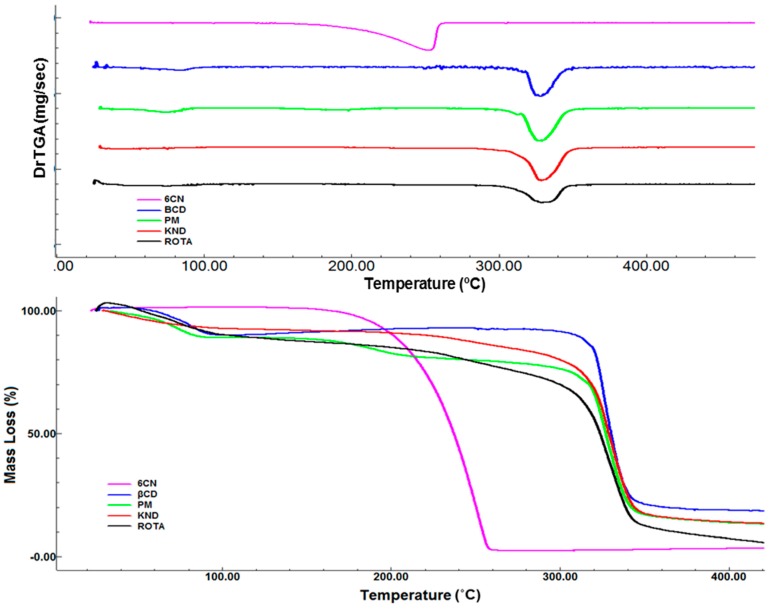
TG/DTG and TGA first derivative curves for 6CN, β-CD, and 6CN/β-CD obtained by PM, KND and ROTA at a heating rate of 10 °C·min^−1^, under dynamic atmosphere of N_2_ (100 mL·min^−1^).

TG/DTG curves for 6CN (Figure 5) shows only a single well-defined event between 146 and 261 °C, with a loss of mass (Δm) of 98.27%. On the other hand, βCD has two events, the first one occurring between 48 and 93 °C with Δm of 10.63% and the second event between 287 and 370 °C with Δm of 72.59%.

TG/DTG curves for 6CN-βCD binary system obtained by PM show four main stages of mass loss. The first event occurs between 30 and 64 °C with Δm of 4.48%, the second occurs between 64 and 90 °C (Δm = 6.42%), the third one occurs between 149 and 209 °C (Δm = 6.88%) and the fourth event occurs between 252 and 379 °C with Δm of 64.92%. The TG curve for KND shows two main stages of mass loss between 31 and 104 °C with Δm of 7.28% and between 199 and 370 °C with Δm of 75.50%. Similar to the TG profile of PM, four stages of mass loss are observed in the TG curve for ROTA. The first event occurs between 29 and 95 °C (Δm = 11.12%), the second one between 95 and 230 °C (Δm = 8.49%), the third event between 230 and 297 °C (Δm = 18.92%) and the fourth one between 297 and 355 °C (Δm = 60.26%).

The TG/DTG curves shown in Figure 5 indicate that while 6CN has only a single well-defined mass loss event, all the 6CN-βCD binary systems show more than one mass loss event, where a larger mass loss is observed near 330 °C which resembles that of βCD’s TGA curve.

It is worth pointing out that the initial thermal event between 48 and 93 °C corresponds to dehydration and it seems to be due to the release of water molecules from the βCD cavity. The event corresponding to the thermal degradation of 6CN occurs around 330 °C for all binary systems. Besides being an indication of interaction with βCD, this higher decomposition temperature is indicative that these binary systems provide a thermal protection for 6CN.

Thermogravimetry is a destructive thermal analysis that monitors the variation of mass of a sample as a function of temperature or time in an environment of controlled temperature and atmosphere [23]. In fact, the improved thermal stability observed for 6CN-βCD might be considered as another indication of association between 6CN and βCD [24].

### 2.5. Fourier Transform Infrared (FTIR)

**Figure 6:**
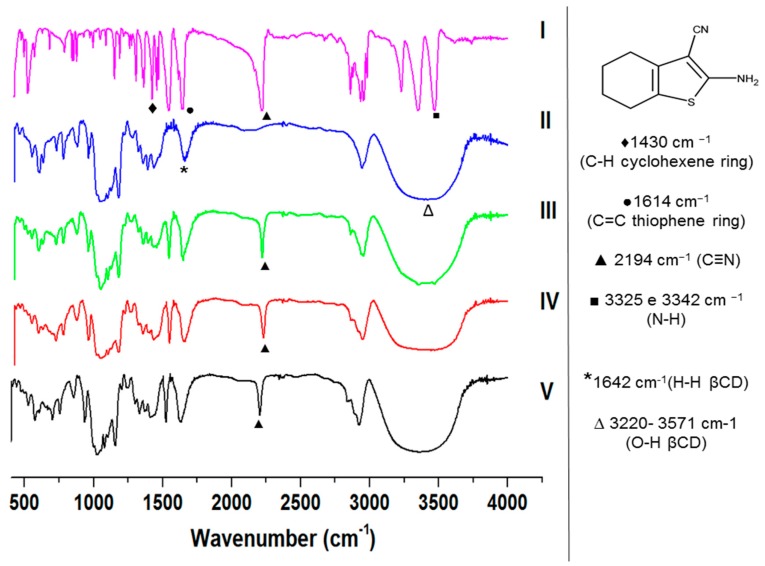
FTIR spectra of 6CN (I), βCD (II), PM (III), KND (IV), ROTA (V) (4000 to 400 cm^−1^ and resolution of 4). The chemical group that correspond to each band is also displayed.

FTIR spectra of 6CN, βCD and 6CN-βCD binary mixtures are shown in Figure 6. The spectrum of 6CN shows two characteristic bands of moderate intensity in the 3325–3342 cm^−1^ region, which relates to the N–H bond of the primary amine. In addition, bands corresponding to the axial deformation of the C≡N triple bond of the isonitrile group at 2194 cm^−1^ and to the axial deformation vibration of the C=C double bond of the thiophene ring at 1614 cm^−1^ are observed. The band at 1430 cm^−1^ corresponds to the asymmetric deformation of the C–H bonds of the cyclohexene ring. The C–S bond of the 6CN thiophene ring has a low intensity band in the region of 700 to 600 cm^−1^ being of little value for the determination of the 6CN structure [25].

The spectrum of βCD shows a broad band between 3571 and 3220 cm^−1^, corresponding to the stretching vibration of the O–H groups (Figure 6). The band at 1642 cm^−1^ corresponds to the stretching vibration of H–H. Other bands were also observed for βCD (2926, 1413, 1366, 1331, 1155, 1085, 1026 and 1000–700 cm^−1^ [26]. In addition, the bands related to C–H and stretching vibrations of CH, CH_2_ and CO (ether-hydroxyl linkages) were observed. The vibrations of the C-H bonds and the skeletal C-C vibrations of the glucopyranose ring are also observed, as previously described elsewhere [27,28].

The FTIR spectra of the binary systems (PM, KND and ROTA) were very similar to that of βCD alone, where the bands related to 6CN were reduced in intensity, shifted or disappeared. In this current study, the absorption band corresponding to the axial deformation of the C≡N of the isonitrile group at 2194 cm^−1^ of 6CN was markedly reduced in intensity in the binary systems. In addition, the absorption band at 1614 cm^−1^ relative to the axial deformation of the C=C double bond of the thiophene ring was shifted in the spectrum of the 6CN-βCD binary systems.

The absorption bands in the 3325–3342 cm^−1^ region due to the N–H binding of the 6CN primary amine were not well identified in the 6CN-βCD binary systems, except for that obtained by PM. On the other hand, a broad band with a maximum absorption between 3571 and 3220 cm^−1^ is observed in the FTIR spectra of the 6CN-βCD, which is similar to that of βCD alone.

The absorption band at 1430 cm^−1^ corresponding to the asymmetric angular deformation of the C–H bonds of the cyclohexene ring was slightly changed in the spectrum of the binary systems with a discrete decrease in intensity.

FTIR has been widely used for the characterization of drug-cyclodextrin association, as it modifies the characteristic bands of the parent drug [29]. In fact, variations in the intensity and position of FTIR bands of both drug and cyclodextrin can provide information about the occurrence of association [19,30]. Our findings indicate that the FTIR spectra of 6CN-βCD binary system resembles that of βCD and therefore, it seems to indicate that 6CN has been associated with βCD, which is corroborated by the disappearance of some of the bands attributed to 6CN. Such finding is usually considered as an indicative of association between drug and cyclodextrin, as previously reported by Liao et al. [21] and Zhang [22].

### 2.6. X-ray Diffraction (XRD)

**Figure 7:**
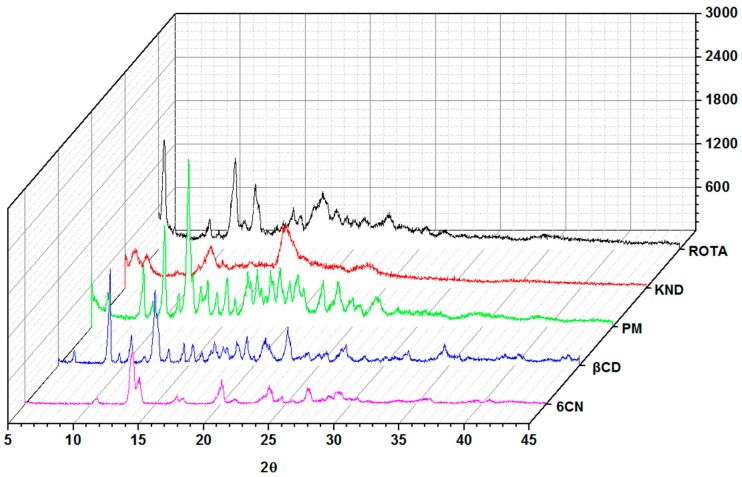
XRD diffratograms of 6CN, βCD and the 6CN-βCD binary systems obtained by PM, KND and ROTA.

The various high intensity crystalline reflections observed in the diffractogram of 6CN indicate that this molecule exists in the crystalline form (Figure 7), as evidenced by the crystalline reflection of greater intensity around 13°, with a diffraction angle of 2θ, in addition to other lower intensity reflections at approximately 20°, 23°, 26° and 29°. The diffractogram of βCD also shows a typical crystalline profile [19], with strong crystalline reflections at 4.4°, 8.9°, 12.3° and 22.6° [31].

On the other hand, the diffractogram of KND shows a predominance of a halo pattern with shifted 6CN crystalline reflections, whereas the sample obtained by ROTA exhibits crystalline reflections even though they are different from the reflections presented by each individual component. The results of the XRD analysis indicate that the binary system prepared by KND method was the one the presented the most amorphous profile, which would probably improve the solubility of 6CN to a greater extent.

The diffractogram of PM exhibits an overlap of crystalline reflections characteristic of both 6CN and βCD, as observed by Wang et al. [19]. Figure 7 shows that the characteristic crystalline reflections of 6CN are not present in the diffractogram of the 6CN-βCD binary systems. In addition, new reflection peaks appear, which suggests that an association between 6CN and βCD has taken place [19]. According to previous reports, βCD shows characteristic peaks at 2θ = 9.06°, 12.6°, 17.06°, 22.54°, 26.96°, 31.91°, 34.70° and 35.95°. Although both the grinding and drying processes may contribute to the amorphization of a drug in a matrix, changes in the drug’s crystalline profile have also been associated with drug-cyclodextrin complexation [32].

### 2.7. Scanning Electron Microscopy (SEM)

**Figure 8:**
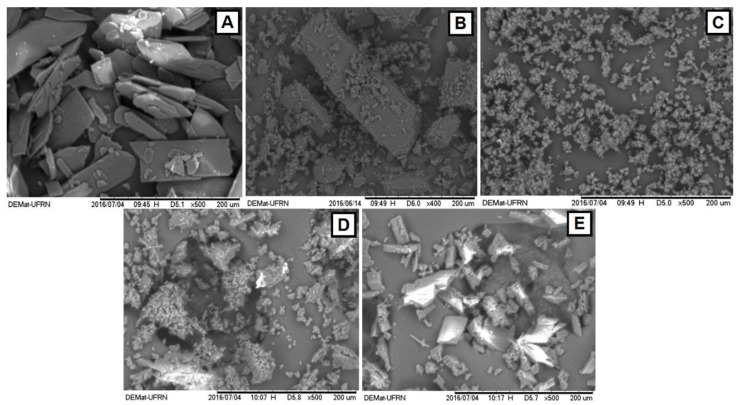
Micrographs of 6CN (**A**), βCD (**B**), and binary systems obtained by PM (**C**), KND (**D**) and ROTA (**E**).

SEM is a qualitative method for evaluating surface texture of solids [19] and therefore, it aims to evaluate the morphology and particle size. The 6CN particles reveal a definite geometric aspect with multilayer-structured crystals of different shapes and sizes (Figure 8), which is attributed to the crystalline character of 6CN as confirmed by the XRD analysis. βCDs are present as irregularly shaped block particles with various dimensions [19] and rhomboid forms, as previously described in the literature [33]. Similar crystalline and homogeneous structures are observed in the micrographs of 6CN-βCD binary systems, where it is possible to observe that such crystals have smaller particle sizes than those of 6CN and βCD alone. The particles of the binary system obtained by ROTA are presented as crystals with block structure.

The micrographs shown in Figure 8 indicate that the morphologies of the binary systems are relatively different from those of individual βCD and 6CN molecules. Since a change in the surface morphology and a reduction in particle size were observed with the binary systems, it suggests that an association between 6CN and βCD has occurred. In fact, the appearance of irregular and amorphous forms, in which both the original morphology and sizes of the individual components have changed, might indicate an association between the drug and cyclodextrin, as reported elsewhere [22,34]. The micrograph of the sample obtained by ROTA exhibited the most crystalline profile of all binary systems. On the other hand, the particles of PM and KND are the ones that presented the highest degree of crystalline change when compared to those of 6CN and βCD.

### 2.8. In Vitro Citotoxiciy Studies

**Figure 9:**
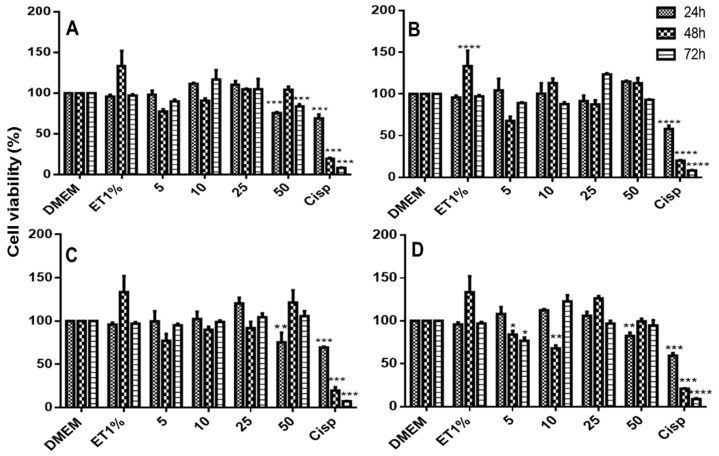
Viability of non-tumoral cells (3T3) as determined by the MTT method after 24, 48, and 72 h of exposure to DMEM (control), ET1% (vehicle), Cisp (50 μM cisplatin as positive control) and 5, 10, 25 and 50 μM of each test sample: 6CN (**A**) and binary systems obtained by PM (**B**), KND (**C**) and ROTA (**D**). Results were expressed as bar charts showing the mean percentage ± SD (*n* = 3 for each experimental group). Statistical analyses were performed by one-way ANOVA with *Tukey-Kramer* post-test using GraphPad Prism version 5.00. A difference in the mean values of of **** *p* < 0.0001, *** *p* < 0.001, ** *p* < 0.01 and * *p* < 0.05 were considered as statistically significant.

**Figure 10:**
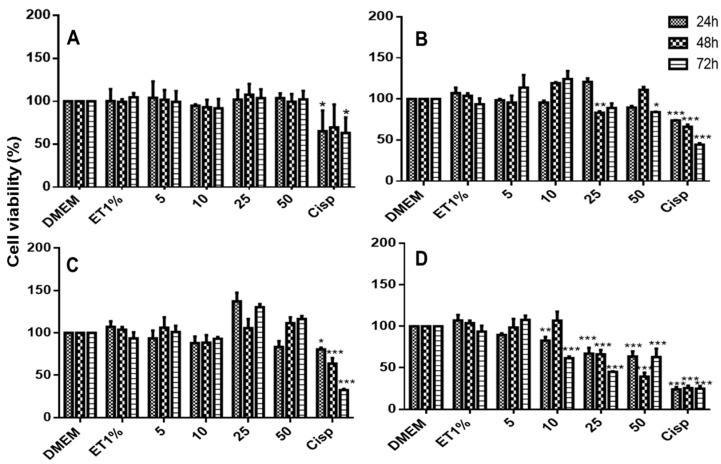
Viability of hepatic adenocarcinoma cells (HepG2) as determined by the MTT method after 24, 48, and 72 h of exposure to DMEM (control), ET1% (vehicle), Cisp (50 μM cisplatin as positive control) and 5, 10, 25 and 50 μM of each test sample: 6CN (**A**) and binary systems obtained by PM (**B**), KND (**C**) and ROTA (**D**). Results were expressed as bar charts showing the mean percentage ± SD (*n* = 3 for each experimental group). Statistical analyses were performed by one-way ANOVA with *Tukey-Kramer* post-test using GraphPad Prism version 5.00. A difference in the mean values of *** *p* < 0.001, ** *p* < 0.01 and * *p* < 0.05 were considered as statistically significant.

**Figure 11:**
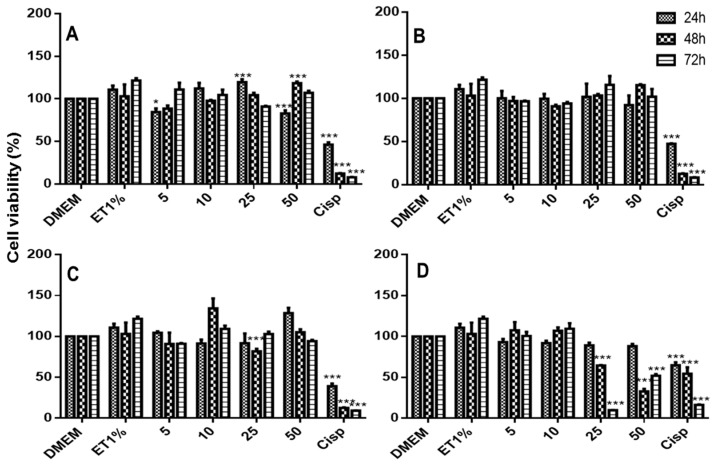
Viability of renal carcinoma cells (786-0) as determined by MTT method after 24, 48, and 72 h of exposure to DMEM (control), ET1% (vehicle), Cisp (50 μM cisplatin as positive control) and 5, 10, 25 and 50 μM of each test sample: 6CN (**A**) and binary systems obtained by PM (**B**), KND (**C**) and ROTA (**D**). Results were expressed as bar charts showing the mean percentage ± SD (*n* = 3 for each experimental group). Statistical analyses were performed by one-way ANOVA with *Tukey-Kramer* post-test using GraphPad Prism version 5.00. A difference in the mean values of *** *p* < 0.001, and * *p* < 0.05 were considered as statistically significant.

The cytotoxicity of 6CN and the binary systems against non-tumoral (3T3) and tumoral cells is depicted in Figure 9, Figure 10 and Figure 11. Although 6CN did not compromise the viability of the non-tumoral cells (Figure 9A), it exhibited cytostatic activity against the tumoral cells HepG2 and 786-0, as observed in Figure 10A and Figure 11A, respectively.

The non-tumoral 3T3 cells demonstrated a proliferative profile over the control group prior to exposure to 6CN and 6CN-βCD binary systems as they showed to be less toxic than the standard drug cisplatin (CISP). Figure 10 and Figure 11 show that 6CN-βCD binary systems were more effective than 6CN, especially towards the 786-0 (Figure 11B–D) and HepG2 (Figure 10B–D) tumor lines.

The 6CN-βCD binary systems at concentrations from 10 to 50 mM showed anti-proliferative effects of 20–80% after 48 h of exposure to 786-0 (Figure 11B–D) and HepG2 (Figure 10B–D) cell lines. Although the different concentrations of 6CN-βCD binary systems showed similar anti-proliferative effects within the first hours of exposure, a marked decrease of cell viability, which was very similar to that of cisplatin, was observed at 48 h as a function of concentration.

It is worth pointing out that not all tumor lines behave in the same way against anticancer agents, where their constitution and particular characteristics may interact through different mechanisms [35]. The results shown in this current study indicate that neither 6CN alone nor associated with βCD significantly affected normal cells, however, they both showed anti-proliferative activity against 786-0 and HepG2 tumor lines, which might indicate a certain selectivity for tumor cell lines, especially after 48 h of exposure, as similarly reported by Aguiar et al. [1] in a study with another 2-aminothiophene derivative.

Although molecular modification is currently the most commonly used strategy to improve the physicochemical and pharmacological properties of drugs [36], this study showed that the association of a novel 2-aminothiophene derivative with cyclodextrin seems to be an efficient alternative to improve their anti-proliferative activity against tumor cells.

## 3. Materials and Methods

The 2-amino-4,5,6,7-tetrahydrobenzo[*b*]thiophene-3-carbonitrile (6CN) was synthesized and supplied by the Laboratory of Drug Synthesis at the State University of Paraíba, while β-cyclodextrin (βCD) was purchased from Sigma Aldrich^®^ (São Paulo, SP, Brazil). All other reagents and solvents were of analytical grade. 

Molecular modeling and docking studies were performed according to the procedure described by Menezes et al. [37]. The chemical structures of 6CN and βCD were designed using Marvin 14.9.8.0, (ChemAxon software; http://www.chemaxon.com, 2014) and saved in sdf format. Geometry optimization and conformational search were performed using Spartan software for Windows 10.0 (Wavefunction Inc., Irvine, CA, USA). The geometry of 6CN and β-CD was initially optimized using the MMFF force field followed by an additional geometry optimization based on the semi-empirical Austin Model 1 (AM1) method. The systematic search method was selected by analyzing 1000 confomers and selecting the 10 lowest energy ones. The dihedral angle was evaluated by rotation according to standard conditions. The optimized geometries and vibrational mode of the lowest energy confomers were calculated using AM1. The docking simulation was performed using Autodock 4.2 software (Scripps Research Institute, La Jolla, CA, USA). Cyclodextrin and 6CN were treated in VEGA ZZ 3.0.0, where the structures of both compounds were saved in pqbqt format. PyRx 0.9 software-X10 was used to analyze the results. A three-dimensional grid box with 50, 44 and 50 points for x, y and z axis, respectively, was created with a spacing of 0.375 Å. The results for each calculation was analyzed in order to obtain the affinity energy values (in Kcal mol^−1^). The Discovery Studio 4.0 software (BIOVIA, San Diego, CA, USA). was used to identify the types of chemical interactions (hydrogen bonds and hydrophobic interactions) and docking interpretation.

The solubility study was performed according to the method described by Higuchi and Connors [13]. Briefly, the saturation of 6CN was achieved in distilled water and buffer solutions with different pH values at 37 °C, where the obtained dispersions were equilibrated by stirring for 3 days and filtered through a 0.45 μm Millipore filter. The saturated solutions were analyzed by UV/visible spectrophotometer at 265 nm. The same procedure was performed in which an excessive amount of 6CN (5 mg) was added to aqueous solutions (5 mL) containing increasing concentrations of βCD (0 mM to 15 mM), where supersaturated solutions were obtained. After the vials were properly sealed, they were subjected to stirring at a constant temperature of 37 °C for 3 days followed by filtration through a 0.45 μm Millipore filter. After proper dilution, the 6CN concentration was spectrophotometrically determined at 265 nm.

The binary systems were prepared by three different methods: physical mixing (PM), kneading (KND) and rotary evaporation (ROTA). For the physical mixture (PM), 6CN and βCD were weighed following the molar ratio of 1:1 and mixed with a mortar and pestle without grinding until a homogeneous mixture was obtained. The resulting PM was kept in a desiccator over silica pellets until use. The binary system prepared by kneading (KND) followed a similar protocol with the same 6CN and βCD molar ratio (1:1), where both were thoroughly mixed with mortar and pestle, after which a hydroalcoholic solution (3:1 ethanol:water) was gradually added and mixed until the formation of a homogeneous paste. Next, the paste was dried in oven at 50 °C for 24 h. After complete drying, the sample was scraped and transferred to a recipient, which was kept in a desiccator over silica pellets until use. For the rotary evaporation method (ROTA), 6CN and βCD (1:1) were previously mixed with mortar and pestle, after which a hydroalcoholic solution (3:1 ethanol:water) was gradually added and mixed until complete homogenization. The resulting mixture was subjected to rotary evaporation under vacuum at 70 °C using rotary evaporator (IKA RV10, GEHAKA,(Staufen, Baden-Wurttemberg, Germany)) at 150 rpm for one hour. The obtained sample was additionally dried at 50 °C in oven for 24 h. Finally, the sample was scraped and transferred to a container, which was kept in a desiccator over silica pellets until use.

Differential scanning calorimetry (DSC) analyzes were carried out using a DSC 50 apparatus (Shimadzu^®^, Tokyo, Japan)) where 3 mg of each sample was hermetically sealed in aluminum crucibles with a heating rate of 10 °C.min^−1^ within the temperature range of 25 to 350 °C under a dynamic atmosphere of N_2_ (50 mL.min^−1^). A blank aluminum crucible was used as reference during the analysis. Prior to the test, enthalpic calibration was performed using indium as standard. The samples submitted to analysis were 6CN, βCD and 6CN-βCD obtained by PM, KND and ROTA.

Thermogravimetric analysis (TGA) were carried out using a Shimadzu^®^ (Tokyo, Japan) DTG 60 thermogravimetric analyzer. About 3 mg of each sample was weighed, sealed in aluminum crucibles and heated at a rate of 10 °C·min^−1^ within the temperature range of 30 to 450 °C under a dynamic atmosphere of N_2_ (100 mL·min^−1^). A blank aluminum crucible was used as reference during the analysis. Prior to the test, the instrument was calibrated with aluminum. The analyzed samples were 6CN, βCD and 6CN-βCD obtained by PM, KND and ROTA. The data were processed using the TA-60 Shimadzu software.

For the Fourier transform infrared (FTIR) analyzes, KBr pellets containing 6CN, βCD and 6CN-βCD obtained by PM, KND and ROTA (2 mg of each sample in 200 mg of KBr) were prepared using a hydraulic press at 10 tons, where the FTIR spectra were obtained (4000 to 400 cm^−1^) using a Shimadzu^®^ IRPrestige-21 spectrometer, with 20 scans and spectral resolution of 4 cm^−1^.

X-ray diffraction (XRD) patterns of 6CN, βCD and 6CN-βCD obtained by PM, KND and ROTA were recorded using a D2 Phaser diffractometer (Bruker, Billerica, MA, USA) for 2θ values ranging from 3° to 45°, using CuKα radiation (λ = 1.54 Å) with a Ni filter, with step of 0.02°, current of 10 mA, voltage of 30 kV and a Lynxeye detector.

The morphology of 6CN, βCD and 6CN-βCD obtained by PM, KND and ROTA was examined by scanning electron microscopy (SEM), where each sample was fixed under double-sided carbon tape and the morphological analysis performed by a TM3000 Tabletop Microscope (HITACHI^®^,(Chiyoda, Tokyo, Japan) ) at 15 kV voltage.

For the in vitro cytotoxicity studies, normal fibroblast cell lines (3T3), hepatic adenocarcinoma (HepG2) and renal carcinoma (786-0) cells were maintained in DMEM-dulbbeco culture medium (Sigma-Aldrich Co, Darmstadt, Germany) and supplemented with 10% FBS at the concentration of 2.5 × 10^4^ mL^−1^, using a 75 cm^3^ culture bottle. Cells were collected every two days using 0.25% trypsin in PBS, centrifuged and re-suspended in fresh culture medium. The cell viability of 6CN and its binary systems with βCD (6CN-βCD) was analyzed through a 3-(4,5-dimethylthiazol-2-yl)-2,5-diphenyltetrazolium bromide (MTT) assay using cell cultures at different concentrations and different time points. Cells were plated at a concentration of 5 × 10^4^ in 96-well microplates containing DMEM medium supplemented with 10% FBS for 24 h, at 36.5 °C and a humidified atmosphere of CO_2_ (5%). After 24 h, the supernatant was removed and 5, 10, 25 and 50 µM of the extract in DMEM medium (10% FBS) were added to the plate in triplicate. After 24, 48 and 72 h of incubation, the entire content within the microplates was discarded and 10% of MTT reagent (Oz Biosciences, San Diego, CA, USA) was added to the wells. After 4 h, the supernatant was discarded and 100 μL of absolute ethanol was added for solubilization of the crystals. The absorbance of each well was measured in an ELISA Reader at 570 nm [38]. Statistical analysis was performed by one-way analysis of variance (ANOVA) and Tukey-Kramer post-test using GraphPad Prism version 5.00 (GraphPad Software, San Diego, CA, USA) at a significance level of 5%.

## 4. Conclusions

In this work, binary systems of 6CN and βCD were prepared using three different methods (physical mixture, kneading and rotary evaporation). The binary systems were systematically analyzed through molecular modeling and phase solubility, as well as characterized by FTIR, XRD, DSC, TG and SEM techniques. The results showed that 6CN was associated with βCD in both aqueous and solid states. Phase solubility studies indicated that 6CN formed stable binary systems with βCD at the 1:1 molar ratio. The characterization analysis showed that the physicochemical characteristics of 6CN were changed after the formation of 6CN-βCD binary systems. According to the results of the MTT assay, 6CN and its binary systems with βCD demonstrated cytostatic and anti-proliferative effects and may be considered as promising anticancer drug candidates. Therefore, the association between 6CN and βCD may be a useful and promising strategy to improve the solubility and antitumor properties of this new chemical entity.
